# Effectiveness and safety of herbal medicine Ukgansan for clinical symptoms in Parkinson's disease: A pilot, randomized, assessor-blinded clinical trial

**DOI:** 10.3389/fneur.2022.1025269

**Published:** 2022-11-10

**Authors:** Chul Jin, Ki-Ho Cho, Seungwon Kwon, Han-Gyul Lee, Tae-Hun Kim, Woo-Sang Jung, Sang-Kwan Moon, Seung-Yeon Cho, Byoung-Kab Kang, Jung-Mi Park, Hi-Joon Park, Chang-Nam Ko

**Affiliations:** ^1^Department of Korean Medicine Cardiology and Neurology, Graduate School, Kyung Hee University, Seoul, South Korea; ^2^Department of Cardiology and Neurology, College of Korean Medicine, Kyung Hee University, Kyung Hee University Medical Center, Seoul, South Korea; ^3^Korean Medicine Clinical Trial Center, Korean Medicine Hospital, Kyung Hee University, Seoul, South Korea; ^4^Department of Cardiology and Neurology, College of Korean Medicine, Kyung Hee University, Kyung Hee University Hospital at Gangdong, Seoul, South Korea; ^5^KM Science Research Division, Korea Institute of Oriental Medicine, Daejeon, South Korea; ^6^Acupuncture and Meridian Science Research Center, College of Korean Medicine, Kyung Hee University, Seoul, South Korea

**Keywords:** Parkinson's disease, Ukgansan, adjunctive therapy, anxiety, quality of life, the 39-item Parkinson's Disease Questionnaire

## Abstract

**Objectives:**

Parkinson's disease (PD) is a neurodegenerative disease in which patients are suffering various symptoms. Previous experimental studies suggested that herbal medicine Ukgansan (UGS) could be beneficial for PD. The aim of this pilot clinical trial was to evaluate the efficacy of UGS for improving clinical symptoms in patients with PD.

**Methods:**

Sixty patients with idiopathic PD were randomly assigned to receive either UGS plus acupuncture or acupuncture alone for 6 weeks. During the trial, all anti-parkinsonian medications were maintained. Subjects were evaluated for various clinical assessments of PD, including the Movement Disorder Society-Sponsored Revision of the Unified PD Rating Scale (MDS-UPDRS) and the 39-item Parkinson's Disease Questionnaire (PDQ-39), until 12 weeks.

**Results:**

In MDS-UPDRS between the groups, no significant time x group interaction was found. In the subgroup analysis of participants with anxiety, a significant time x group interaction was found in the PDQ-39 domain of mobility (*P* = 0.007), activities of daily living (*P* = 0.042), and the PDQ-39 summary index (*P* = 0.048). In addition, *post-hoc* analysis in participants with anxiety showed a significant decrease in the domains of mobility (*P* = 0.001) and activities of daily living (*P* = 0.013) at week 7. There were no adverse events associated with UGS.

**Conclusion:**

The additional administration of UGS has the potential to significantly improve the quality of life of PD patients with anxiety. In order to create more definitive evidence, clinical trials with more rigorous methodologies should be conducted in future.

**Clinical trial registration:**

http://cris.nih.go.kr, identifier: KCT0003444.

## Introduction

Parkinson's disease (PD) is the second most frequent neurodegenerative disorder, with a prevalence of 2–3% in the elderly population (1). In 2015, 6.2 million people worldwide were diagnosed with PD, and the number is projected to be over 12 million by 2040 (2).

Parkinson's disease (PD) is characterized by a lack of dopamine within basal ganglia due to cell loss of dopaminergic neurons in the substantia nigra, resulting in motor symptoms such as bradykinesia, resting tremor, rigidity, and postural instability. Therefore, the gold-standard treatment of PD is to alleviate parkinsonian symptoms with drugs that increase dopamine concentrations (levodopa) or directly stimulate dopamine receptors (dopamine agonists) (3). However, long-term use of anti-PD medications reduces their effectiveness and causes side effects such as neuropsychiatric symptoms and motor complications. In addition, PD patients are suffered from not only motor symptoms, but also numerous non-motor symptoms, such as depression, fatigue, sleep disorder, cognitive impairment, autonomic dysfunction, and pain which might start in the prodromal phase of PD (1, 3). Thus, managing these various symptoms of PD requires a variety of therapeutic options in addition to conventional therapy.

Complementary alternative medicine (CAM) treatments for PD include meditation, qigong, yoga, dance, massage, acupuncture, and herbal medicine (4). Among them, acupuncture has been used as an effective therapeutic option in patients with PD in eastern Asia (5). Previous clinical trials have shown that acupuncture has the clinical efficacy to improve motor symptoms and some non-motor symptoms in patients with PD (6). Furthermore, acupuncture has also been found to protect dopaminergic neurons from degeneration by altering the neurotransmitter balance in the basal ganglia circuit and reducing oxidative stress, inflammation, and apoptosis (7).

As another option, that herbal medicine as an adjunct therapy can help improve motor and non-motor symptoms of PD (8, 9). Ukgansan (UGS, Yokukansan in Japanese, Yigansan in Chinese) is an herbal medicine formula that may benefit the treatment of PD and that has been shown to protect dopaminergic neurons and to supplement dopamine concentration (10, 11). However, the therapeutic role of UGS in patients with PD is debatable. UGS has improved neuropsychiatric symptoms but not motor function in clinical trials for PD (12, 13). Therefore, an exploratory pilot clinical trial to determine whether UGS helps improve clinical symptoms of PD is needed. The present pilot study explored the efficacy and safety of UGS for improving clinical symptoms in patients with PD.

## Materials and methods

### Study design

This study was a single-centered, randomized controlled, assessor-blinded, active-controlled, parallel-group, pilot clinical trial conducted at the Kyung Hee University Korean Medicine Hospital (Seoul, Republic of Korea) between December 2018 and November 2020. The protocol was registered with the Clinical Research Information Service (CRIS, KCT0003444) and approved by the Institutional Review Board of Kyung Hee University Korean Medicine Hospital (KOMCIRB-170717-HR-02).

Participants were recruited through advertisements, which are poster ads on bulletin boards of the hospital and the subway. This pilot trial had three consecutive periods: screening period (week 0), intervention period (weeks 1–6), and observation period (weeks 7–12). All prospective participants were questioned by phone by the clinical research coordinator (CRC) to check their medications and PD duration before being scheduled for a screening visit (Visit 0). At the screening visit, patients who voluntarily agreed to participate in the clinical trial were evaluated on the inclusion and exclusion criteria, including blood sampling and electrocardiogram (ECG).

**Figure 1:**
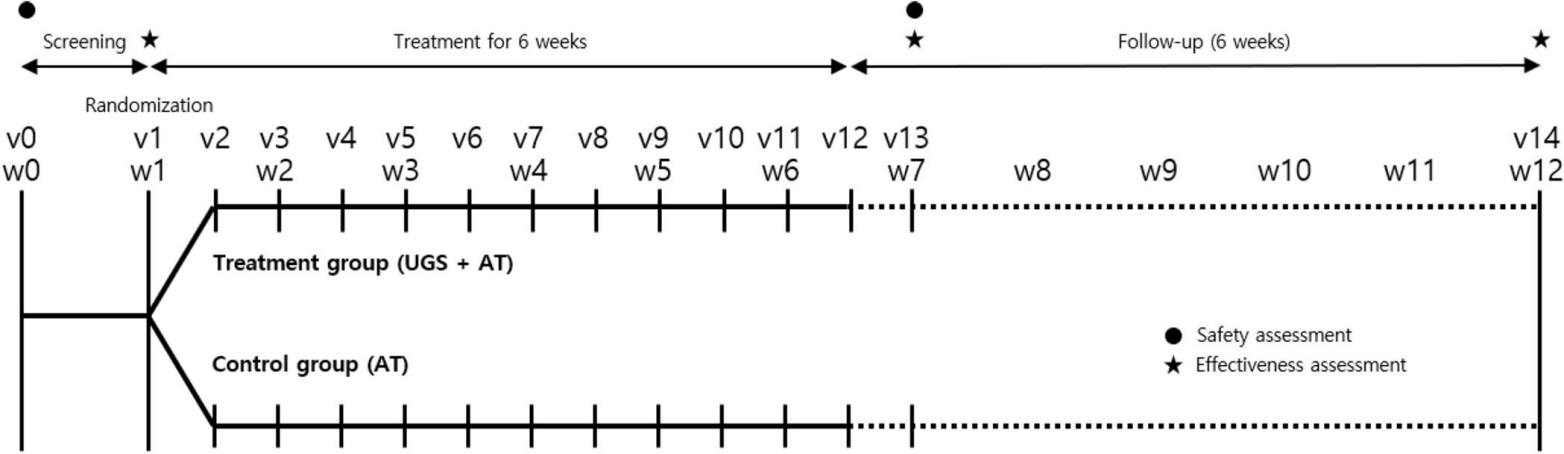
Timeline of study. The vertical lines indicate the time points of the actual visit for trial, and the dotted lines show the observation period after the intervention. Clinical effectiveness was assessed with the Movement Disorder Society-Unified Parkinson's Disease Rating Scale (MDS-UPDRS), the modified Hoehn and Yahr staging scale (H–Y scale), Schwab and England Activities of Daily Living (SE-ADL) Scale, Berg balance test (BBT), Timed Up and Go test (TUG), 39-item Parkinson's Disease Questionnaire (PDQ-39), and five-level version of European Quality of Life Scale Five-Dimensional Questionnaire (EQ-5D-5L) score. Self-reported adverse events and measured vital signs were evaluated at each visit. Electrocardiogram and blood sampling were performed before and after treatment for safety assessment. v, visit; w, week; UGS, Ukgansan; AT, acupuncture.

At week 1, baseline measurements were taken on eligible participants. After that, they were randomly assigned to one of two groups, the control group (only acupuncture + medications related to Parkinson's disease) or the treatment group (UGS + acupuncture + medications related to Parkinson's disease) in a 1:1 ratio. From that day on, participants in both groups maintained medications related to Parkinson's disease and were treated for acupuncture two times a week for 6 weeks. The treatment group was also prescribed UGS for the same period. Measurements for evaluations were taken at week 7 and week 12 (Figure 1).

If the following situation occurred while participating in the study, it was treated as a withdrawal:

1. Violation of the inclusion or exclusion criteria after enrollment,
2. Withdrawal of consent by the participants (or their legal representative),
3. Request for discontinuance of the trial or refusal to receive intervention by the participants (or their legal representative),
4. Loss to follow-up,
5. Serious adverse events (e.g., hospitalization or life-threatening events, etc.) or exacerbation of illnesses that made it difficult to continuous participation,
6. Less than 80% compliance of clinical trial medication (UGS) administration and/or acupuncture treatment,
7. Violation of the clinical trial protocol,
8. Other inappropriate conditions for study participation as judged by the investigator.

### Participants

Participants who met the following criteria were selected as subjects of the present study.

#### Inclusion criteria

1. Males or females aged 45–80 years,
2. Patients with idiopathic Parkinson's disease taking levodopa for more than 5 years (without anti-cholinergic drugs),
3. Hoehn and Yahr stages 2–3,
4. Patients without cognitive impairment who have voluntarily agreed to participate.

#### Exclusion criteria

1. Patients diagnosed with secondary parkinsonism or atypical parkinsonian syndrome,
2. Diseases that might affect the administration or absorption of drugs (e.g., dysphagia, clinically severe digestive disorders, galactose intolerance, Lapp lactase deficiency, or glucose–galactose malabsorption),
3. Previous history of severe heart disease (myocardial infarction, heart failure, etc.),
4. Patients receiving neurosurgical treatment (e.g., deep brain stimulation),
5. Patients with glaucoma,
6. Patients who were diagnosed or treated for cancer within 5 years,
7. Chronic alcohol consumption or drug abuse,
8. Patients with a history of allergy to the test drug (UGS),
9. Patients with liver disease or kidney disease (aspartate aminotransferase (AST), alanine aminotransferase (ALT), blood urea nitrogen (BUN), or creatinine >x3 of normal upper limit),
10. Women who were planning pregnancy, pregnant, or breastfeeding,
11. Participation in other clinical trials within 30 days,
12. Patients who were judged by the investigator to be unsuitable for participation in the trial due to psychiatric symptoms, medical illness, laboratory findings, etc.

### Interventions

All participants maintained medications related to Parkinson's disease (levodopa, dopamine agonists, monoamine oxidase B (MAO-B) inhibitors, catechol-O-methyltransferase (COMT) inhibitors, amantadine, etc.) except for anti-cholinergic drugs, which had been taken as before.

#### Control group

The acupuncture therapy was performed two times a week for 6 weeks on participants in the control arm. Acupuncture was conducted by a Korean medical doctor with more than 7 years of clinical expertise in internal medicine. The STRICTA checklist included instructions on how to practice acupuncture (Table 1) (14).

**Table 1:** Revised STRICTA^*^ checklist.

1. Acupuncture rationale	1a) Manual acupuncture 1b) Based on a systematic review^**5**^ and expert consensus 1c) Unacceptable
2. Details of needling	2a) 23 points 2b) GV20, bilateral EX-HN5, GB20, LI4, TE5, LI10, LI11, GB34, ST36, GB39, LR3, GB41^**†**^ 2c) 5-10mm 2d) De-qi 2e) Manual stimulation 2f) 20 minutes 2g) Sterilized stainless steel needle (diameter 0.25mm, length 40mm, Dongbang Medical co., Seongnam, South Korea)
3. Treatment regimen	3a) 12 sessions 3b) two times a week for 6 weeks
4. Other components of treatment	4a) The purpose of this study was to verify the additional effects of UGS when used in conjunction with acupuncture. As a result, during the intervention time, the treatment group received UGS two times a day, and the anti-Parkinson's medicine remained the same. 4b) The study was conducted in an outpatient treatment room at a single Korean medicine hospital. Before deciding to enroll in the clinical study, the patient was given all pertinent information.
5. Practitioner background	5) All acupuncture procedures were performed by one Korean Medicine Doctor with more than 7 years of clinical experience.
6. Control interventions	6a) Based on the systematic review^**5**^ and expert consensus 6b) Same as acupuncture procedure of the treatment group

* Standards for Reporting Interventions in Clinical Trials of Acupuncture.

† The terminology was followed as the WHO Standard Acupuncture Point Locations in the Western Pacific Region.

#### Treatment group

Participants in the treatment group received the same acupuncture procedure as the control group and additional administration of UGS. UGS, an herbal extract granule, was produced and packaged by KYUNGJIN PHARM.CO.LTD. (Icheon, South Korea), one of the Korean Good Manufacturing Practice (KGMP)-certified companies, for this clinical trial under the approval of the Ministry of Food and Drug Safety's Investigational New Drug (IND). The composition of UGS is described in Table 2. At visits 1, 5, and 9, participants were provided with individually packed UGS (14 days + an extra 2 days). UGS was provided by an independent pharmacist in a separate place, and participants were required to take that medicine with water at 10 a.m. and 5 p.m. for 6 weeks.

**Table 2:** Composition of Ukgansan^*^.

**Scientific name**	**Herbal name**	**Amount (*g*)**
*Atractylodes lancea* De Candolle	Atractylodis Rhizoma	4
*Poria cocos* Wolf	Poria Sclerotium	4
*Magnolia officinalis* Rehder et Wilson	Magnoliae Cortex	4
*Poncirus trifoliata* Rafinesque	Ponciri Fructus Immaturus	4
*Cnidium officinale* Makino	Cnidii Rhizome	3
*Uncaria sinensis* (Oli.) Havil	Uncariae Ramulus Et Uncus	3
*Angelica gig*as Nakai	Angelicae Gigantis Radix	3
*Bupleurum falcatum* Linné	Bupleuri Radix	2
*Glycyrrhiza uralensis* Fisher	Glycyrrhizae Radix et Rhizoma	1.5

* The Ukgansan used in this study was modified by adding Magnoliae Cortex and Ponciri Fructus Immaturus to original Ukgansan.

### Outcomes

#### Primary outcome

The primary outcome of this study was a change in the Movement Disorder Society-Unified Parkinson's Disease Rating Scale (MDS-UPDRS) total score after treatment (week 7 minus baseline), between the two groups (15).

#### Secondary outcomes

The secondary outcomes included MDS-UPDRS part I–IV, trends of changes in MDS-UPDRS, the modified Hoehn and Yahr staging scale (H–Y scale), Schwab and England Activities of Daily Living (SE-ADL) Scale, Berg balance test (BBT), Timed-up-and-go (TUG) test, 39-item Parkinson's Disease Questionnaire (PDQ-39) including the PDQ-39 summary index (PDQ-39 SI), and five-level version of European Quality of Life Scale Five-Dimensional Questionnaire (EQ-5D-5L) score (16, 17). All these measured outcomes were compared between the two groups with the changes in difference from baseline.

#### Safety assessment

Vital signs and adverse events were measured at each visit. ECG and blood sampling were performed at the screening visit and week 7. Blood sampling includes the white blood cell count (WBC), red blood cell count (RBC), hemoglobin (Hb), hematocrit (Hct), platelet count, BUN, creatinine, AST, ALT, glucose, and electrolytes (Na, K, Cl).

### Sample size calculation

This clinical trial was to verify the additional improvement in MDS-UPDRS total score following the administration of herbal medicine UGS. The effect size was calculated from the previous study which was conducted to validate the effect of herbal medicine and performed by using original UPDRS (18). The pooled standard deviation of the UPDRS total score at post-treatment in a previous similar study was 16.64. And a minimal clinical important difference (CID) on the UPDRS total score was 4.3 points (19). Assuming the mean difference of 4.3 and the standard deviation of 16.64, the calculated effect size was 0.258. We calculated the sample size using stepped rules of thumb for pilot sample size, using a two-tailed type I error rate of 5% and a power of 90%. As a result, each group required 25 participants (20). Sixty participants, 30 in each group, were recruited, anticipating a 15% drop-out rate similar to the previous study (21).

### Randomization, allocation concealment, and blinding

An independent statistician generated random numbers by block randomization using STATA (version 4.2; StataCorp LLC, Texas, USA). The random assignment code was placed in opaque sealed envelopes and delivered to the Kyung Hee University Korean Medicine Hospital. The investigator opened the consecutive numbered sealed envelope in front of the participant whose screening criteria were satisfied and assigned them to the intervention or control group. All opened envelopes were kept safely and separately.

The design of this study does not include placebo as a control; therefore, the participants and practitioners cannot be blinded. However, the separate assessor who was responsible for evaluating efficacy and safety was blinded to prevent access to the allocation results.

### Statistical analysis

At baseline assessments, continuous data were expressed as means and standard deviations and analyzed with either the two-sample *t*-test or the Wilcoxon rank-sum test. Categorical variables were represented as frequencies or percentages, and the Chi-square test or Fisher's exact test was used to assess them.

The effectiveness analysis was preferentially performed using the full analysis set (FAS), including participants who had been treated more than once after participating in the clinical trial. If necessary, the per-protocol set (PPS) was performed, including participants who had completed the clinical trial plan. In the FAS analysis, missing data were handled with the last-observation-carried-forward (LOCF) analysis imputation method.

The MDS-UPDRS score changes in primary and secondary outcomes, between baseline (before the randomization) and week 7 (1 week after 6 weeks of treatment), were compared between the two groups using either the two-sample *t*-test or the Wilcoxon rank-sum test. A repeated measures analysis of variance (ANOVA) was used to examine the trend of changes in the MDS-UPDRS score between groups depending on the time. The modified H–Y scale evaluated at two endpoints was classified as an improvement, no change, or aggravation, compared with baseline. And the improvement ratio between groups was conducted using the Chi-square test or Fisher's exact test. The other secondary outcomes, comparing the change scores between groups at weeks 7 and 12, were analyzed using the same methods as the primary outcome. The significance of the statistical values between groups at each time point was evaluated using an independent *t*-test as a *post-hoc* analysis.

The safety of treatment was assessed using the safety analysis set (SAS) method, which included all subjects who had been treated at least once. And the Chi-square test or Fisher's exact test was used whether there were any differences in the incidence ratio between the groups.

This pilot clinical trial was conducted with a two-sided test to determine significance at the 5% level (α = 0.05). In the *post-hoc* test, the Bonferroni correction was applied to test the significance (statistically significant when *p* < 0.016). All statistical analyses were performed using the IBM SPSS Statistics for Windows, version 25 (IBM Corp., Armonk, NY, USA).

## Results

### Baseline characteristics

**Figure 2:**
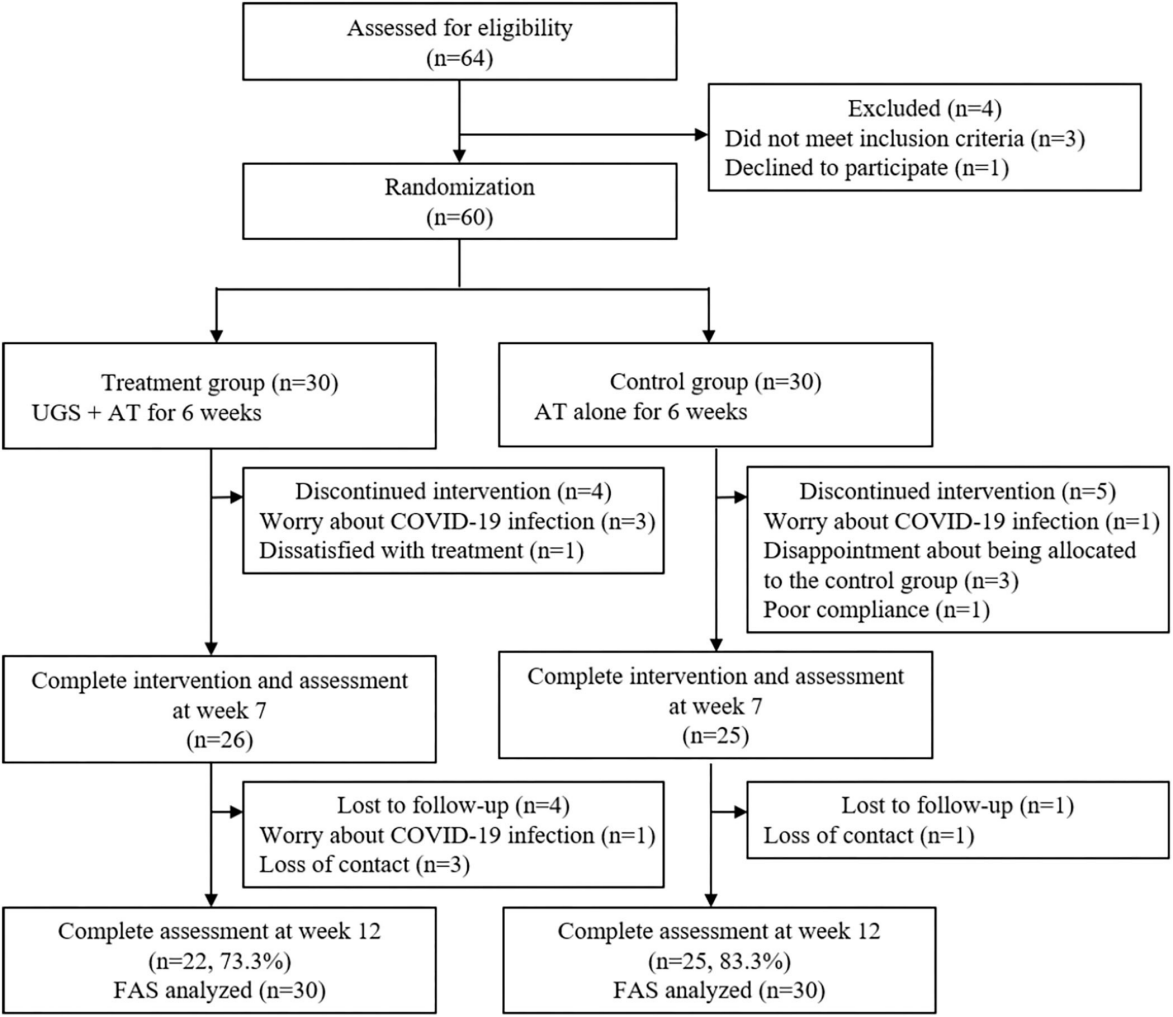
Study flow diagram. UGS, Ukgansan; AT, acupuncture; COVID-19, coronavirus disease 2019.

A total of 64 patients were screened for eligibility. Of these 64, three did not meet the inclusion criteria, one withdrew one's consent, and the remaining 60 subjects were randomly assigned to the treatment group and the control group, 30 in each group. During the 6-week treatment period, four participants withdrew from the treatment group, and five dropped out of the control group. And four patients in the treatment group and one in the control group did not show up for a follow-up visit after 6 weeks. As a result, 22 (73.3%) of the treatment participants and 25 (83.3%) of the control participants completed the final evaluation (Figure 2).

The demographic and baseline characteristics of both groups are listed in Table 3. There were no statistically significant differences in demographics, clinical features, or outcome variables.

**Table 3:** Demographic and baseline characteristics.

**Characteristics**	**Treatment group** ** (*n* = 30)**	**Control group** ** (*n* = 30)**	***P* value**
Age, mean (SD), years	66.87 (8.04)	64.27 (7.18)	0.192
Male, n (%)	15 (50.00)	19 (63.33)	0.297
BMI, mean (SD), kg/m^2^	22.51 (2.56)	23.92 (2.96)	0.053
**Employment status**			
Employed, *n* (%)	3 (10.00)	1 (3.33)	
Unemployed, *n* (%)	27 (90.00)	29 (96.67)	0.612
**Education**			
Primary school, *n* (%)	17 (56.67)	8 (26.67)	
Middle school, *n* (%)	7 (23.33)	13 (43.33)	
High school, *n* (%)	3 (10.00)	7 (23.33)	
College or higher, *n* (%)	3 (10.00)	2 (6.67)	0.074
PD history, mean (SD), years	9.27 (3.77)	9.10 (3.36)	0.857
Levodopa dosage, mean (SD), mg/day	626.67 (303.35)	691.67 (274.99)	0.336
MDS-UPDRS total score, mean (SD)	57.47 (20.35)	58.63 (26.67)	0.850
MDS-UPDRS part I, mean (SD)	12.40 (6.08)	13.53 (7.82)	0.533
MDS-UPDRS part II, mean (SD)	14.37 (7.54)	15.10 (8.47)	0.724
MDS-UPDRS part III, mean (SD)	27.77 (13.71)	26.07 (14.12)	0.638
MDS-UPDRS part IV, mean (SD)	2.93 (3.34)	3.93 (3.84)	0.286
**Modified Hoehn–Yahr stage**			
Stage 2, *n* (%)	25 (83.33)	23 (76.67)	
Stage 2.5, *n* (%)	1 (3.33)	2 (6.67)	
Stage 3, *n* (%)	4 (13.33)	5 (16.67)	0.794
BBT, mean (SD)	53.00 (3.24)	51.73 (4.95)	0.245
TUG, mean (SD), sec	8.56 (2.80)	9.49 (6.58)	0.484
SE-ADL, mean (SD), %	82.00 (10.64)	83.00 (12.08)	0.735
**Domains of PDQ-39, mean (SD), %**			
Mobility	27.58 (19.78)	29.17 (23.22)	0.777
Activities of Daily Living	29.31 (19.50)	25.56 (24.34)	0.513
Emotional well-being	29.86 (23.85)	31.67 (24.29)	0.772
Stigma	24.17 (16.47)	28.96 (23.81)	0.369
Social support	27.22 (22.52)	20.28 (19.41)	0.206
Cognition	29.79 (21.88)	25.63 (21.17)	0.457
Communication	23.61 (19.34)	23.06 (24.04)	0.922
Bodily discomfort	28.89 (23.03)	31.94 (21.23)	0.595
PDQ-39 SI, mean (SD), %	27.55 (16.11)	27.03 (19.32)	0.910
EQ-5D Index score, mean (SD)	0.72 (0.11)	0.70 (0.15)	0.440
EQ-5D VAS, mean (SD), mm	71.70 (13.75)	73.23 (16.38)	0.696

BMI, body mass index; PD, Parkinson's disease; MDS-UPDRS, the Movement Disorder Society-Unified Parkinson's Disease Rating Scale; BBT, Berg balance test; TUG, Timed Up and Go test; SE-ADL, Schwab and England Activities of Daily Living Scale; PDQ-39, 39-item Parkinson's Disease Questionnaire; PDQ-39 SI, PDQ-39 summary index; EQ-5D, five-level version of European Quality of Life Scale Five-Dimensional Questionnaire; VAS, Visual analog scale.

*P* value was calculated using independent *t*-test or Wilcoxon rank-sum test.

### MDS-UPDRS

At the repeated measures analysis of variance used to determine the trend of change in MDS-UPDRS between the groups, no significant time x group interaction was found. In the total MDS-UPDRS score and part III total score, however, only the time effect was significant, indicating that the two scores tended to decrease until the 12-week observation period (Table 4).

**Table 4:** The trend of changes during 12 weeks in the MDS-UPDRS score between groups depending on the time using repeated measures analysis of variance.

**Variable**	**Time**	**Treatment group**	**Control group**	**Source**	**F value**	***P* value**
		**(*n* = 30)**	**(*n* = 30)**			
Total MDS-UPDRS score	Baseline	57.47 ± 20.35	58.63 ± 26.67	Group	0.008	0.930
	Week 7	53.03 ± 18.55	53.30 ± 24.01	Time	8.406	0.001
	Week 12	52.13 ± 21.89	49.23 ± 25.20	Time x group	0.683	0.489
MDS-UPDRS Part I score	Baseline	12.40 ± 6.08	13.53 ± 7.82	Group	0.169	0.682
	Week 7	11.90 ± 5.64	12.30 ± 6.57	Time	2.076	0.138
	Week 12	11.63 ± 4.82	11.93 ± 6.75	Time x group	0.286	0.717
MDS-UPDRS Part II score	Baseline	14.37 ± 7.54	15.10 ± 8.47	Group	0.217	0.643
	Week 7	13.60 ± 6.20	15.00 ± 9.29	Time	0.225	0.799
	Week 12	14.07 ± 8.06	14.57 ± 8.12	Time x group	0.204	0.816
MDS-UPDRS Part III score	Baseline	27.77 ± 13.71	26.07 ± 14.12	Group	0.777	0.382
	Week 7	24.80 ± 15.05	22.13 ± 12.58	Time	10.889	<0.001
	Week 12	23.97 ± 14.17	19.50 ± 13.62	Time x group	0.771	0.447
MDS-UPDRS Part IV score	Baseline	2.93 ± 3.34	3.93 ± 3.84	Group	1.274	0.264
	Week 7	2.73 ± 3.56	3.87 ± 4.13	Time	2.025	0.137
	Week 12	2.47 ± 2.78	3.23 ± 3.71	Time x group	0.187	0.830

MDS-UPDRS, the Movement Disorder Society-Unified Parkinson's Disease Rating Scale.

Values are presented as mean ± standard deviation.

In both groups, the MDS-UPDRS total score and MDS-UPDRS part I–IV score measured at week 7 decreased from baseline. However, the difference in total MDS-UPDRS score from baseline at week 7, which was a primary outcome, did not show any significance between the two groups (95% CI: −0.82 to 6.22; *P* = 0.801). Similarly, there were no significant differences in MDS-UPDRS part I–IV scores between the two groups after treatment (Table 5).

**Table 5:** The difference in MDS-UPDRS scores between groups from baseline to week 7.

	**Treatment group**	**Control group**	**Mean difference**	**95% confidence**	***P* value**	**Effect size**
	**(*n* = 30)**	**(*n* = 30)**		**interval (CI)**		
Total MDS-UPDRS score	−4.43 ± 14.04	−5.33 ± 13.50	−0.90	−8.02 to 6.22	0.801	0.065
Part I score (non–motor symptom)	−0.50 ± 4.97	−1.23 ± 5.07	–	–	0.868	0.145
Part II score (motor symptom)	−0.77 ± 5.79	−0.10 ± 5.17	–	–	0.747	0.122
Part III score (motor examination)	−2.97 ± 8.53	−3.93 ± 8.75	−0.97	−5.43 to 3.50	0.666	0.111
Part IV score (motor complications)	−0.20 ± 2.62	−0.07 ± 2.53	–	–	0.760	0.050

MDS-UPDRS, the Movement Disorder Society-Unified Parkinson's Disease Rating Scale.

Values are presented as mean ± standard deviation.

*P* value was calculated using independent *t*-test or Wilcoxon rank-sum test.

Effect size was calculated using Cohen's D.

### H–Y scale

At the two outcome points, weeks 7 and 12, no significant difference in H–Y scale changes was observed between the two groups (*P* = 1.00 and *P* = 0.240, respectively) (Table 6).

**Table 6:** Comparison of H–Y scale improvement rates between groups.

	**Change of H–Y scale**	**Treatment group**	**Control group**	***P* value**
		***n*** **(%)**		
Week 7–baseline	Improvement	10 (33.3)	10 (33.3)	1.00
	No change	19 (63.3)	20 (66.7)	
	Aggravation	1 (3.3)	0 (0)	
Week 12–baseline	Improvement	12 (40.0)	18 (60.0)	0.240
	No change	17 (56.7)	11 (36.7)	
	Aggravation	1 (3.3)	1 (3.3)	

H–Y scale, Modified Hoehn–Yahr stage scale.

*P*-value was calculated by Fisher's exact test.

### Other outcomes (BBT, TUG, SE-ADL, PDQ-39, and EQ-5D-5L)

There were no significant interaction effects evaluated by repeated measures ANOVA in all measured outcomes. Between the two groups, there were no significant differences in the BBT, TUG, SE-ADL, PDQ-39 SI, PDQ-39 domains, EQ-5D index score, and EQ-5D VAS (Table 7).

**Table 7:** Comparison of other outcomes between two groups.

	**Week**	**Treatment group**	**Control group**	**Source**	*P* value^*^	***P* value^†^**
		**(*n* = 30)**	**(*n* = 30)**			
BBT	0	53.00 ± 3.24	52.07 ± 4.59	Group	0.665	–
	7	53.27 ± 2.97	53.33 ± 4.33	Time	0.007	0.071
	12	53.63 ± 3.31	53.27 ± 4.54	T × G	0.236	0.374
TUG	0	8.56 ± 2.80	9.49 ± 6.58	Group	0.521	–
	7	7.71 ± 2.56	8.55 ± 6.56	Time	<0.001	0.800
	12	7.90 ± 2.89	8.63 ± 6.66	T × G	0.844	0.985
SE-ADL	0	82.00 ± 10.64	83.00 ± 12.08	Group	0.680	–
	7	84.00 ± 13.03	82.00 ± 12.15	Time	0.232	0.146
	12	85.67 ± 8.58	83.33 ± 13.22	T × G	0.320	0.365
PDQ-39 SI	0	27.55 ± 16.11	27.03 ± 19.32	Group	0.884	–
	7	25.24 ± 15.78	26.62 ± 18.22	Time	0.620	0.359
	12	26.04 ± 16.95	27.07 ± 19.47	T × G	0.768	0.264
Domains of PDQ-39						
Mobility	0	27.58 ± 19.78	29.17 ± 23.22	Group	0.287	–
	7	22.42 ± 19.60	32.08 ± 23.20	Time	0.676	0.030^†^
	12	24.25 ± 18.17	29.17 ± 22.93	T × G	0.111	0.306
Activities of	0	29.31 ± 19.50	25.56 ± 24.34	Group	0.687	–
Daily Living	7	26.81 ± 20.17	27.36 ± 23.13	Time	0.676	0.182
	12	30.42 ± 22.51	27.22 ± 24.07	T × G	0.530	0.522
Emotional	0	29.86 ± 23.85	31.67 ± 24.29	Group	0.592	–
Wellbeing	7	25.14 ± 20.19	28.47 ± 24.12	Time	0.106	0.867
	12	26.11 ± 21.24	30.00 ± 25.22	T × G	0.850	0.928
Stigma	0	24.17 ± 16.47	28.96 ± 23.81	Group	0.572	–
	7	25.83 ± 17.43	26.67 ± 23.21	Time	0.414	0.224
	12	22.92 ± 18.95	25.83 ± 24.44	T x G	0.539	0.441
Social support	0	27.22 ± 22.52	20.28 ± 19.41	Group	0.404	–
	7	24.72 ± 21.27	19.44 ± 20.57	Time	0.632	0.600
	12	24.17 ± 24.21	23.61 ± 22.11	T × G	0.289	0.060
Cognition	0	29.79 ± 21.88	25.63 ± 21.17	Group	0.616	–
	7	27.29 ± 21.49	25.63 ± 18.30	Time	0.748	0.370
	12	28.54 ± 22.72	27.08 ± 20.52	T × G	0.748	0.517
Communication	0	23.61 ± 19.34	23.06 ± 24.04	Group	0.889	–
	7	23.33 ± 19.99	23.06 ± 23.02	Time	0.857	0.836
	12	24.72 ± 19.14	23.33 ± 25.37	T × G	0.933	0.395
Bodily discomfort	0	28.89 ± 23.03	31.94 ± 21.23	Group	0.529	–
	7	26.39 ± 23.27	30.28 ± 17.84	Time	0.447	0.768
	12	27.22 ± 24.26	30.28 ± 21.27	T × G	0.942	0.927
EQ-5D Index score	0	0.72 ± 0.11	0.70 ± 0.15	Group	0.462	–
	7	0.73 ± 0.13	0.73 ± 0.12	Time	0.101	0.833
	12	0.73 ± 0.13	0.69 ± 0.16	T × G	0.417	0.561
EQ-5D VAS	0	71.70 ± 13.75	73.23 ± 16.38	Group	0.840	–
	7	68.50 ± 15.60	70.30 ± 19.10	Time	0.094	0.882
	12	73.70 ± 16.25	72.67 ± 18.88	T × G	0.694	0.420

BBT, Berg balance test; TUG, Timed Up and Go test; SE-ADL, Schwab and England Activities of Daily Living Scale; PDQ-39, 39-item Parkinson's Disease Questionnaire; PDQ-39 SI, PDQ-39 summary index; EQ-5D, five-level version of European Quality of Life Scale Five-Dimensional Questionnaire; VAS, Visual analog scale; T, Time; G, Group.

Values are presented as mean ± standard deviation.

* *P* value was calculated using repeated measures ANOVA.

† *P* value was calculated comparing differences from baseline (Δ score) between groups by independent *t*-test or Wilcoxon rank-sum test.

### PDQ-39 domains and summary index in anxiety group

All subjects who answered no to item 21 (anxious) on the PDQ-39 were eliminated, and only those with anxiety were subjected to subgroup analysis. In a subgroup of anxiety, *post-hoc* analysis was performed on the PDQ-39. In this subgroup analysis, Cronbach's alpha value for PDQ-39 was 0.926, which showed high reliability.

In a repeated measures ANOVA to investigate changes between the two anxiety groups over time, the PDQ-39 SI score, mobility domain, and activities of daily living domain showed a significant time x group interaction effect (*P* = 0.048, *P* = 0.007, and *P* = 0.042, respectively, Table 8). And comparing the differences from baseline between the two groups, the treatment group with anxiety showed a significant improvement in the domains of mobility and activities of daily living compared with the control group with anxiety at week 7 (*P* = 0.001, *P* = 0.013, respectively, Table 8).

**Table 8:** Comparison PDQ-39 domains and summary index between groups with anxiety.

	**Week**	**Treatment group with**	**Control group with**	**Source**	*P* value*	***P* value^†^**
		**anxiety (*n* = 17)**	**anxiety (*n* = 20)**			
PDQ-39 SI	0	31.93 ± 15.80	27.67 ± 17.61	Group	0.847	–
	7	25.17 ± 14.94	28.89 ± 17.10	Time	0.312	0.056
	12	26.37 ± 16.70	29.93 ± 18.50	T × G	0.048	0.020
Domains of PDQ-39						
Mobility	0	31.32 ± 19.12	29.63 ± 19.96	Group	0.190	–
	7	19.41 ± 17.49	35.63 ± 21.32	Time	0.365	0.001
	12	23.09 ± 14.91	31.13 ± 21.38	T × G	0.007	0.038
Activities of	0	35.29 ± 18.81	24.79 ± 21.44	Group	0.651	–
Daily Living	7	26.47 ± 19.65	29.58 ± 22.21	Time	0.695	0.013
	12	30.64 ± 21.29	29.38 ± 23.70	T × G	0.042	0.024
Emotional	0	37.75 ± 20.96	34.17 ± 19.00	Group	0.927	–
Wellbeing	7	28.92 ± 20.49	31.25 ± 21.27	Time	0.084	0.434
	12	30.15 ± 21.93	33.13 ± 23.12	T × G	0.410	0.365
Stigma	0	26.10 ± 18.25	30.31 ± 22.88	Group	0.460	–
	7	25.00 ± 17.54	29.38 ± 22.31	Time	0.465	0.981
	12	22.43 ± 17.12	27.81 ± 24.37	T × G	0.969	0.921
Social support	0	32.84 ± 24.20	19.58 ± 18.19	Group	0.320	–
	7	28.92 ± 21.67	21.25 ± 20.32	Time	0.761	0.341
	12	26.47 ± 26.72	28.33 ± 21.01	T × G	0.057	0.004
Cognition	0	35.29 ± 22.53	27.50 ± 21.69	Group	0.961	–
	7	24.63 ± 18.42	27.81 ± 19.29	Time	0.161	0.040
	12	27.21 ± 19.51	30.94 ± 21.12	T × G	0.059	0.024
Communication	0	25.49 ± 17.30	20.00 ± 21.19	Group	0.491	–
	7	24.02 ± 20.17	20.83 ± 19.21	Time	0.801	0.502
	12	25.98 ± 19.07	22.08 ± 23.77	T × G	0.898	0.774
Bodily discomfort	0	31.37 ± 23.85	35.42 ± 23.71	Group	0.184	–
	7	24.02 ± 19.07	35.42 ± 18.90	Time	0.253	0.222
	12	25.00 ± 21.45	36.67 ± 22.52	T × G	0.166	0.151

PDQ-39, 39-item Parkinson's Disease Questionnaire; PDQ-39 SI, PDQ-39 summary index; T, Time; G, Group.

*P* value was calculated using repeated measures ANOVA.

† *P* value was calculated comparing differences from baseline (Δ score) between groups by Mann–Whitney's *U* test.

### Safety

For the safety analysis, a total of 60 subjects were included. There were no serious adverse events in either group, and the trial was not terminated due to any adverse events. A subject in the treatment group reported mild dizziness (*n* = 1, 3.33 %). After acupuncture treatment, that participant experienced mild dizziness, which went away after a brief rest. At week 7, a subject in the control group (*n* = 1, 3.33 %) reported an increase in WBC. WBC elevation was thought to be caused by a common cold, and after re-examination at a follow-up visit, it returned to normal. Aside from that, there were no adverse events with clinical abnormalities, vital signs, blood samples, or ECG.

## Discussion

In comparison with the control group, the treatment group did not reveal significant changes in the MDS-UPDRS score at week 7 and other outcomes at weeks 7 and 12. In subgroup analysis of anxiety participants, a significant time x group interaction was found in the PDQ-39 domain of mobility, activities of daily living, and the PDQ-39 summary index. In the *post-hoc* analysis of PDQ-39 on patients with anxiety who responded to item 21 (anxious) of PDQ-39, significant differences between the two groups were detected in the domains of mobility and activities of daily living at week 7.

In line with these real-world clinical situations of Korea, we planned to apply acupuncture treatment in the same regimen for both the test group and the control group. Acupuncture is the most common treatment for patients with PD who visit a Korean medicine hospital for outpatient, followed by herbal medicine (22). At actual clinical sites of Korean medicine, using herbal medicine alone without acupuncture for PD is unusual unless there are extraordinary cases, such as needle phobia. There are two reasons: The first is that acupuncture is an effective and safe treatment for PD and the second is that it is covered by Korean national health insurance, so the cost burden is minimal. Therefore, acupuncture is performed in almost all patients with PD by Korean medical doctors, but herbal medicine is administered as necessary.

The pattern identification in traditional East Asian medicine, which is a pattern of symptom manifestation that varies between individuals, is used in clinical herbal medicine to make diagnoses and prescribe medicine. And UGS was traditionally used to treat neurosis, insomnia, night crying, and irritability in children. UGS had been shown in previous studies to improve neuropsychiatric symptoms such as aggression, agitation, and anxiety in patients with PD or Alzheimer's disease (12, 13, 23–26). According to an animal study, the anti-anxiety effect of UGS might be mediated by serotonin (upregulation of 5HT1A and downregulation of 5HT2A) and glutamate release (glutamate transport by increasing express glutamate transporter mRNA) (27). Despite these scientific findings, herbal medicine may not have the same effect on everyone. Treatment that focuses on symptoms rather than diseases, such as traditional methods, might be more effective (28). In Japan, 15% of RCTs using herbal medicine included a pattern of symptom manifestation diagnosis (29). Therefore, clinical trials in combination with the traditional diagnosis are thought to be a better way to demonstrate traditional East Asian medicine's characteristics. A real-world trial was conducted in this manner, with positive results (30). Therefore, for the above reason, it is considered that the treatment group was more effective on the PDQ-39 than the control group in the *post-hoc* test of the subgroup with anxiety.

Anxiety is a common non-motor symptom in patients with PD. Concerns about PD, experiences in an “off” state, and neurotransmitter reduction are all known to cause anxiety (31). According to Hannah et al., anxiety is more important than depression in predicting quality of life of the patients with PD (32). In this study, PD patients with anxiety based on item 21 of PDQ-39 were included in the *post-hoc* analysis. Item 21, anxious, was known to have a strong relationship with the State-Trait Anxiety Inventory (STAI), the most widely used anxiety self-assessment tool (33). The STAI had also been used in several studies to assess anxiety in patients with Parkinson's disease, and it was reliable and valid (34, 35). As a result, we divided the patients into anxiety subgroups based on their responses to item 21.

In the control group with anxiety, there was no significant improvement in PDQ-39 after acupuncture + medications related to Parkinson's disease. Acupuncture's efficacy in alleviating non-motor symptoms in PD is less well known than its effect on alleviating motor symptoms. It mainly showed significant effects on depression or insomnia (6), but in PDQ-39, previous research has shown a variety of results following acupuncture treatment, making it difficult to make a definitive conclusion (36). And the negative effect may have had a role in this trial because the control group was not given placebo medicines.

This study aims to find out whether there was effectiveness and safety in the administration of UGS compared with the control group. However, there was no additional change in clinical symptoms of Parkinson's disease in the treatment group compared to the control group. Only the quality of life of participants with anxiety was significantly affected. Similar results were found in previous studies in which UGS was administered to patients with Parkinson's disease. Although UGS reduced the Neuropsychiatric Inventory (NPI) score, it did not affect the UPDRS III (motor examination) or the H–Y scale (12, 13). Furthermore, Uncaria in UGS exhibited a similar effect as Aripiprazole, an antipsychotic drug that can act as a partial dopamine agonist and cause drug-induced parkinsonism (37). When taken as above, UGS may not be able to aid in the improvement of motor function. However, UGS has been reported to increase dopamine levels with similar effects to catechol-O-methyltransferase (COMT) inhibitors, as well as protect dopamine neurons from neurotoxicity and improve drug-induced parkinsonian symptoms (10, 11, 38, 39). Moreover, UGS was also known to ameliorate dyskinetic movement caused by levodopa (40). Taking these heterogeneous findings into account, more research into the efficacy of UGS is thought to be necessary.

There are some limitations to the present study. First, in this study, the participants in the control group could not be blinded. For this reason, there may be a placebo effect in the treatment group. Second, a 6-week treatment period may not be long enough to obtain the result. Clinical trials examining the effectiveness of herbal medicines in PD had a period of almost 12 weeks or more (9). As a result, the 6-week period may have been insufficient to see the intact effect of UGS. However, even in a short period, the administration of UGS showed significant effectiveness in the anxiety group. Finally, there were mixed cases where “on” and “off” were not the same at the time of measurement. In this trial, “on” and “off” states were analyzed together. However, the “off”-state participant was few, and it is estimated that their impact will not be significant.

Despite the limitations mentioned above, this research had some advantages. This study was a pilot clinical trial to verify the efficacy of herbal medicine UGS based on recent experimental results. The study found that the clinical effectiveness of UGS on QoL examined with PDQ-39 was estimated to be significantly larger in patients with PD and anxiety. The findings of this study can be used as a base for large-scale clinical trials on the efficacy of UGS in the treatment of PD in future.

## Data availability statement

The original contributions presented in the study are included in the article/supplementary material, further inquiries can be directed to the corresponding author.

## Ethics statement

The studies involving human participants were reviewed and approved by the Institutional Review Board of Kyung Hee University Korean Medicine Hospital. The patients/participants provided their written informed consent to participate in this study.

## Author contributions

CJ, K-HC, and SK conceived the objectives of the study. CJ acquired the data. CJ, B-KK, H-JP, and SK analyzed the data and performed the statistical analysis, which was discussed with W-SJ and T-HK. CJ, K-HC, and SK wrote the first draft of the manuscript. CJ, W-SJ, H-GL, S-YC, S-KM, C-NK, H-JP, and SK co-drafted the final version. K-HC and SK supervised the study. All authors critically revised the manuscript and have approved the final manuscript.

## Funding

This work is supported by the grant from the Traditional Korean Medicine R&D Program and funded by the Ministry of Health and Welfare through the Korea Health Industry Development Institute (KHIDI, HB16C0051) and the National Research Foundation of Korea (NRF), funded by the Ministry of Science and ICT (No. 2022M3A9B6017813).

## Conflict of interest

The authors declare that the research was conducted in the absence of any commercial or financial relationships that could be construed as a potential conflict of interest.

## Publisher's note

All claims expressed in this article are solely those of the authors and do not necessarily represent those of their affiliated organizations, or those of the publisher, the editors and the reviewers. Any product that may be evaluated in this article, or claim that may be made by its manufacturer, is not guaranteed or endorsed by the publisher.
